# Application of random forest based on semi-automatic parameter adjustment for optimization of anti-breast cancer drugs

**DOI:** 10.3389/fonc.2022.956705

**Published:** 2022-07-22

**Authors:** Jiajia Liu, Zhihui Zhou, Shanshan Kong, Zezhong Ma

**Affiliations:** ^1^ College of Science, North China University of Science and Technology, Tangshan, China; ^2^ Hebei Engineering Research Center for the Intelligentization of Iron Ore Optimization and Ironmaking Raw Materials Preparation Processes, North China University of Science and Technology, Tangshan, China; ^3^ The Key Laboratory of Engineering Computing in Tangshan City, North China University of Science and Technology, Tangshan, China; ^4^ Hebei Key Laboratory of Data Science and Application, North China University of Science and Technology, Tangshan, China; ^5^ Tangshan Intelligent Industry and Image Processing Technology Innovation Center, North China University of Science and Technology, Tangshan, China

**Keywords:** anti-breast cancer, parameter optimization, random forest, xgboost, bioactivity

## Abstract

The optimization of drug properties in the process of cancer drug development is very important to save research and development time and cost. In order to make the anti-breast cancer drug candidates with good biological activity, this paper collected 1974 compounds, firstly, the top 20 molecular descriptors that have the most influence on biological activity were screened by using XGBoost-based data feature selection; secondly, on this basis, take pIC50 values as feature data and use a variety of machine learning algorithms to compare, soas to select a most suitable algorithm to predict the IC50 and pIC50 values. It is preliminarily found that the effects of Random Forest, XGBoost and Gradient-enhanced algorithms are good and have little difference, and the Support vector machine is the worst. Then, using the Semi-automatic parameter adjustment method to adjust the parameters of Random Forest, XGBoost and Gradient-enhanced algorithms to find the optimal parameters. It is found that the Random Forest algorithm has high accuracy and excellent anti over fitting, and the algorithm is stable. Its prediction accuracy is 0.745. Finally, the accuracy of the results is verified by training the model with the preliminarily selected data, which provides an innovative solution for the optimization of the properties of anti- breast cancer drugs, and can provide better support for the early research and development of anti-breast cancer drugs.

## 1 Introduction

At the present, stroke, ischemic heart disease and other cardiovascular diseases, as well as malignant tumors represented by breast cancer have become the main cause of premature death in our population, seriously threatening human health. Global incidence rate and mortality associated with breast cancer have been increasing (1), and breast cancer has officially replaced lung cancer as the number one cancer worldwide (2), and its incidence rate among women’s cancers worldwide is as high as 24.2%, becoming one of the most common cancers in women (3–5), which seriously affects women’s health (6). Estrogen receptor is a hormone receptor and an effective nonstandard RNA binding protein. It is a biomarker of breast cancer and affects the choice of endocrine therapy for breast cancer. It has a very important role in the process of breast development. It is considered as an important target for the treatment of breast cancer and plays an important role in the treatment of breast cancer. Therefore, compounds that can antagonize the activity of ERα may be drug candidates for the treatment of breast cancer.

Active compounds are compounds that can have an effect on disease sites in the human body, or compounds with more pronounced pharmacological effects and clear structures, which are widely used in research fields such as cancer, stem cells and immunity. Molecular descriptors refer to the measurement of the properties of molecules in a certain aspect, which can be either the physical and chemical properties of molecules or the numerical indicators derived from various algorithms according to the molecular structure. The target is a kind of biological macromolecule which has pharmacodynamic function and can be acted by drugs. The biological activity of a compound refers to its ability to bind, inhibit or activate the target. The higher the biological activity, the stronger the ability of the compound. Quantitative Structure-Activity Relationship aims to establish the quantitative relationship between the physiological activities or some properties of a series of compounds and their physical and chemical property parameters or structural parameters through reasonable mathematical statistical methods.

Currently, in the research and development of cancer drugs, the process of screening and developing new drugs through experiments is very slow and requires a lot of manpower and material resources, how to effectively and quickly select drugs to treat breast cancer and improve the therapeutic effect has become an important topic in the research of cancer drugs. In order to save time and cost, the method of establishing compound activity prediction models are usually used to screen potentially active compounds. The specific method is as follows: for a target related to disease, collect a series of compounds acting on the target and their biological activity data, and then take a series of analytical structural descriptors as independent variables and the biological activity value of compound as dependent variables to build a Quantitative Structure-Activity Relationship (QSAR) model of the compound. Then using the model to predict new molecules with better bioactivity, or to guide the structural optimization of existing active compounds.

This paper presents a four-part study on the optimization of anti-breast cancer drug properties. (1) In section 1, this paper analyzes the research that has been completed in related fields, the application of artificial intelligence algorithm and describes the research content of this paper. (2) In section 2, the theoretical basis of the used algorithm is described. (3) In section 3, the data set, preprocessing of the data as well as the analysis of the results are presented, including the XGBoost-based data feature selection results and prediction results of Quantitative Structure-Activity Relationship (QSAR) model. (4) In section 4, a summary of the work done throughout the text is presented.

## 2 Related work

B Zhao (7) considered the independence, coupling and correlation of bioactivity descriptors to screen the most potentially valuable bioactivity descriptors, and then used an optimized back propagation neural network pair to make predictions, and used a gradient boosting algorithm to verify the pharmacokinetics and safety of the screened bioactivity descriptors, and the results showed that the bioactivity descriptors screened by this method not only fit the non-linear relationship of activity well, but also accurately predicted their pharmacokinetic characteristics and safety. The results showed that the screened bioactivity descriptors could not only fit the nonlinear relationship of activity, but also accurately predict the pharmacokinetic characteristics and safety, with an average accuracy of 89.92 ~ 94.80%. S Leya, P N Kumar (8) established a deep learning based cancer drug screening model to predict the activity in the GDB13 data set after confirming the importance of synergy between an effective mimetic drug or compound and its target, and achieved good results in identifying anti-cancer drugs with improved performance metrics. B Xu (9) constructed a QSAR model based on three traditional neural network models (BP neural network, Elman neural network and wavelet neural network) and a neural network model improved by optimization algorithm (SSA-BP neural network), and the results showed that the BP neural network can predict the biological activity of compounds more accurately, and the optimized model can further improve the predictive performance of the BP neural network, which can help to better screen efficient compound molecules and guide the structural optimization of existing active compounds and the development of quality breast cancer drugs. X Liu, W Zhang, W Zheng, et al. (10) considered that the low content of traditional drug screening platforms limits the process of drug evaluation, so it proposed a micropatterned co-culture-based high content (µCHC) platform to study neuronal cancer cell interactions and drug screening, and finally obtained a high efficiency and fidelity of clinical cancer treatment by screening drug candidates or drug combinations through the µCHC system. Y Zhu, T Brettin, Y A Evrard, et al. (11) extended the classical transfer learning framework by integration and demonstrated its general utility with a gradient advancement model and two deep neural networks for three representative prediction algorithms, and finally tested the integrated transfer learning framework on an *in vitro* drug screening benchmark dataset, and the results showed that the established framework extensively improved the prediction algorithms in prediction applications prediction performance. Z Xiong, D Wang, X Liu, et al. (12) used a new graph neural network structure (Attentive FP), which uses a graph attention mechanism to achieve learning from relevant drugs in a data set, and experimental results showed that Attentive FP achieved state-of-the-art prediction performance in various datasets. P Wongyikul, N Thongyot, P Tantrakoolcharoen, et al (13) developed an had screening protocol using Gradient Boosting Classifier machine learning model and screening parameters to identify HAD prescription error events from drug prescriptions. The experimental results show that machine learning plays an important role in screening and reducing HAD prescription errors and has potential benefits. D FernándezLlaneza, S Ulander, D Gogishvili, et al. (14) proposed a Siamese recurrent neural network model (SiameseCHEM) based on bidirectional long-term and short-term memory structure with self attention mechanism, which can automatically learn the discriminant features from the SMILES representation of small molecules. Then it is trained with random SMILES strings, which proves that it is robust to binary or classification tasks of biological activity. M Kumari, N Subbarao (15) proposed a new deep learning based approach to implement virtual screening with convolutional neural network architecture as a way to predict the inhibitory activity of 3CLpro against unknown compounds during SARS-CoV virtual screening. Experimental results show that their proposed convolutional neural network model can prove useful for the development of novel target-specific anti-SARS-CoV compounds. A Abdo, M Pupin (16) proposed a turbine prediction model using nearest neighbor structure to improve prediction accuracy in order to study how to use learning data to enhance prediction model. The experimental results show that Turbo prediction can improve the prediction quality of the traditional prediction model. For heterogeneous data sets, it can predict with minimal computational cost without additional efforts of users. A Gupta, H Zhou (17) accelerated the screening of drugs by opening a machine-learning driven large-scale virtual screening pipeline in order to handle the growing library of drug-like compounds and to separate true positives from false positives. K Carpenter, A Pilozzi, X Huang (18) created a virtual screener for protein kinase inhibitors and achieved prediction of IC50 values for target compounds by transforming and feeding the data as input into two majority-invariant recurrent neural networks (RNN).

With the progress of science and technology, the development of artificial intelligence technology is changing with each passing day. Its application fields are very wide, and it can be effectively applied to all fields of production and life. Of course, the application advantages of artificial intelligence are very obvious. More and more enterprises are committed to the R&D and application of artificial intelligence. With the deepening of research, the application rate and popularity of artificial intelligence technology are also gradually increasing. For example, artificial intelligence can be applied to online learning. By applying artificial intelligence technology and educational psychology theory, personalized online learning resource recommendation schemes can be designed to improve students’ learning outcomes (19). Artificial intelligence can also be applied to multi-objective optimization. For example, in order to improve the existing technology of proton exchange membrane fuel cell (PEMFC), multi-objective optimization based on artificial intelligence can be adopted to facilitate the design and application of PEMFC (20). To address the issue of geographic emergency evacuation of vulnerable population groups, multi-objective planning can be used to improve the safety of evacuees during the natural disaster preparation phase and to ensure timely evacuation from areas expected to be affected by major natural disasters (21). Artificial intelligence can also be applied to the field of transportation. With highly interconnected road networks placing higher demands on road safety and efficiency, intelligent transportation systems have received widespread attention. Artificial intelligence technology can provide various support for road routing and traffic congestion management, and can effectively support intelligent transportation systems (22). Artificial intelligence can also be applied to several fields in the medical field, such as neural disease prediction and modeling, bioinformatics, surgery, physical rehabilitation, medical robot and hospital clinical data management (23). The most basic is the grass-roots medical institutions, which are the first line of defense for the health of grass-roots residents. Its informatization construction is an important means to realize the modernization of medical services. Artificial intelligence technology can promote the informatization of grass-roots medical institutions, so as to optimize the process of medical treatment, improve the service capacity of high-quality medical resources and reduce costs (24). Artificial intelligence technology can be applied to the financial field. With the continuous expansion of the scale, quantity and scope of international trade and the increase of trade complexity and uncertainty, artificial intelligence technology can predict and select international trade and play an important role in the healthy development of international trade (25). The Financial Stability Board (FSB) also released the development of artificial intelligence and machine learning in the financial service market and their impact on financial stability. Artificial intelligence and machine learning can certainly strengthen financial supervision (26). Artificial intelligence techniques can also be applied in industry, for example, the surface roughness induced by grinding operations can affect the corrosion resistance, wear resistance, and contact stiffness of ground parts, which can be predicted using artificial intelligence algorithms, helping to provide real-time feedback control of grinding parameters for the purpose of reducing production costs (27). Artificial intelligence techniques can also protect the network from data transmission, for example, P Rani, Kavita, S Verma, et al. (28) proposed a new update routing protocol combining the advantages of artificial bee colony, artificial neural network and support vector machine techniques as a way to protect the network from black hole attacks. Artificial intelligence techniques can also be applied in the field of scheduling, for example, to solve the scheduling problem of CDT trucks, M Dulebenets (29) proposed a new adaptive multiplicative modal algorithm, which can assist in the correct planning of CDT jobs. Artificial intelligence techniques can also improve algorithms; for example, to address the problem of Gaussian noise impeding the unbiased aggregation capability of GNN models, W Dong, M Wozniak, J Wu, et al., (30) proposed a method that uses principal component analysis to retain the aggregated true signal from adjacent features and simultaneously removes filtered Gaussian noise to achieve a more advantageous denoising capability.

The main contributions of this paper are as follows: XGBoost is used for data feature selection so as to select the 20 molecular descriptors with the most significant impact, and then the 20 molecular descriptors screened are used as input variables and the pIC50 value as output variables from the perspective of the compound molecular descriptors, and four machine learning algorithms, namely Gradient-enhanced regression, XGBoost regression, Support vector machine, and Random Forest regression are used for comparison. The results of Random Forest regression, XGBoost regression and Gradient-enhanced regression are preliminarily screened out to be good. Then the Semi-automatic parameter adjustment method is used to adjust the parameters of the three algorithms, and subsequently the most appropriate algorithms is selected to determine the core algorithm of the prediction model as a way to predict the IC50 and pIC50 values. The highest accuracy rate of 74.5% is finally obtained for Random Forest regression, and the Random Forest algorithm is considered to be the core algorithm.

## 3 Theoretical foundation

### 3.1 XGBoost-based data feature selection

Feature selection refers to the selection of some effective features from the original features to reduce the dimensionality of the data set (31). XGBoost is an integrated learning model that can fit the residuals of the previous tree by generating a new tree in successive iterations and its accuracy increases with the number of iterations (32), which can be effectively used for classification and regression (33).

XGBoost is an improvement of the Gradient boosting algorithm by using Newton’s method when solving the extrema of the loss function, Taylor expansion of the loss function to the second order, and additionally a regularization term is added to the loss function. The objective function at training time consists of two parts, the first part is the Gradient boosting algorithm loss and the second part is the regularization term. The loss function is defined as:


$$\text{L}\left(\emptyset \right)=\sum_{\text{i}=1}^{\text{n}}\left(y_{\text{i}}^{'},{\text{y}}_{\text{i}}\right)+\sum_{\text{k}}\Omega \left({\text{f}}_{\text{k}}\right)$$

Where n is the number of training function samples, l is the loss for a single sample, which is assumed to be a convex function, $y_{\text{i}}^{'}$ is the predicted value of the model for the training samples, and *y_i_* is the true label value of the training samples.

The regularization term defines the complexity of the model:


$$\Omega \left(\text{f}\right)=\gamma \text{T}+\frac{1}{2}\lambda {\left\|\text{w}\right\|}^{2}$$

Where λ and λ are manually set parameters, w is a vector formed by the values of all leaf nodes of the Decision tree, and T is the number of leaf nodes.

### 3.2 Random forest

Random Forest is a supervised learning algorithm which tends to find the best grouping features recursively (34). The “forest” it builds is an integration of Decision tree, which is mostly trained using Bagging methods. The Bagging method uses randomly selected training data with playback and then constructs a classifier, and finally combines the learned models to increase the overall effect.

The growth of the tree in the Random Forest algorithm introduces additional randomness to the model. Unlike Decision tree where each node is partitioned into the best features that minimize the error, in a Random Forest we randomly select features to construct the best partition. Thus, when you are in a Random Forest, consider only the random subset used to segment the nodes, or even make the tree more random by using a random threshold on each feature instead of searching for the best threshold as in a normal Decision tree. This process yields a wide range of diversity and usually leads to better models, and the Random Forest algorithm proceeds as follows:

The input is the sample set D={ (x_1_,y_1_),(x_2_,y_2_),⋯,(x_m_,y_m_) } and the number of weak classifier iterations T.

The output is the final strong classifier f(x).

1) For t=1,2,⋯,T: divided into two steps. a) The training set is randomly sampled for the tth time, and a total of m times are taken to obtain the sampling set *D_t_* containing m samples. b) Train the first t Decision tree model *G_t_* (*x*) with the sample set *D_t_*. When training the nodes of the Decision tree model, select a part of the sample features among all the sample features on the nodes, and choose an optimal feature among these randomly selected part of the sample features to do the left and right subtree partitioning of the Decision tree.

2) In case of classification algorithm prediction, the category or one of the categories with the most votes cast by T weak learners is the final category. In case of regression algorithms, the value obtained by arithmetic averaging of the regression results obtained by T weak learners is the final model output.

### 3.3 Gradient-enhanced regression tree

Gradient-enhanced regression tree(GBR) is a nonparametric machine learning method based on propulsion strategies and Decision trees (35), whose basic idea is to use regression trees as weak learners and replace a single strong learner with a superposition of multiple weak learners. We train multiple layers of weak classifiers for the same training set, and each layer uses the training set to train a weak classification model, from which we obtain the prediction results. We then determine the weights that should be reassigned to each sample based on whether the samples in the training set are correctly classified and the accuracy of the overall classification, and train a classifier for the next layer with the new data set after the modified weights. This training is continued until there are few misclassified samples, and finally the classifiers of each layer with weight assignments are fused together so that the final decision classifier is composed down.

### 3.4 Support vector machine

Support vector machine (SVM) is a supervised machine learning that can deal with classification and regression problems (36), the basic idea is to find the optimal classification hyperplane that completely separates the two classes of samples in the original space in the linearly divisible case and to use kernel methods in the nonlinear case to solve problems that are nonlinear in low-dimensional space as linearly integrable problems in high-dimensional space (37). Delineating the hyperplane can be defined as a linear equation:


$$w^{T}x+b=0$$

Where w={ w_1_,⋯,w_d_ } is a normal vector that determines the direction of the hyperplane, d is the number of eigenvalues, x is the sample to be trained, and b is the displacement term that determines the distance between the hyperplane and the origin.

Suppose P(x_1_,⋯,x_n_) is a point in the training sample, where *x_i_* denotes the ith feature variable of that sample. Then the formula for the distance from the point to the hyperplane is:


$$\text{d}=\frac{\left|{\text{w}}_{1}*{\text{x}}_{1}+{\text{w}}_{2}*{\text{x}}_{2}+\cdots +{\text{w}}_{\text{n}}*{\text{x}}_{\text{n}}+\text{b}\right|}{\sqrt{{\text{w}}_{1}^{2}+{\text{w}}_{2}^{2}+\cdots +w_{\text{n}}^{2}}}=\frac{\left|W^{\text{T}}*\text{X}+\text{b}\right|}{\left\|\text{W}\right\|}$$

Where ‖W‖ is the parametrization of the hyperplane and the constant b is the intercept in the linear equation.

In the case that the hyperplane is determined, the full support vector can be found and then the hyperplane interval can be calculated. The next step is to determine w and b so that the interval is maximum. This is an optimization problem whose objective function can be written as:


$$\text{arg max}\left\{\text{min}\left(\text{y}\left(w^{\text{T}}+\text{b}\right)\right)*\frac{1}{\left\|\text{W}\right\|}\right\}$$

Where y denotes the label of the training sample point and its value is -1 or 1, and y(w^T^+b) denotes the distance. If the training sample points are in the positive direction of the hyperplane, then y(w^T^+b) is a positive number, and the opposite is a negative number. This is an optimization problem with constraints and can usually be solved by the Lagrange multiplier method.


$$\text{L}\left(\text{w},\text{b},\text{a}\right)=\frac{1}{2}*\left\|\text{w}\right\|-\sum_{\text{i}=1}^{\text{n}}{\text{a}}_{\text{i}}\left({\text{y}}_{\text{i}}\left(\text{w}*\text{x}+\text{b}\right)-1\right)$$

This optimization algorithm gives us *a* ^*^, and then we can solve for w and b according to *a* ^*^. The purpose of the classification is to find the hyperplane, i.e., the “decision plane”.

### 3.5 Semi-automatic parameter adjustment

Semi-automatic parameter adjustment is a parameter adjustment method combining manual parameter adjustment and grid search. For different algorithms, it has different sequence of parameter adjustment steps. Taking XGBoost parameter adjustment as an example, the process is as follows:

1) First grid search n_ estimators parameter, other parameters take fixed values;

2) Take the optimization result in (1) and add it to the parameter setting, and grid search min_child_ weight and max_ depth two parameters;

3) Take the optimization result in (1)(2) and add it to the parameter setting, and grid search gamma parameter;

4) Take the optimization result in (1)~(3) and add it to the parameter setting, and grid search subsample and colsample_bytree two parameters;

5) Take the optimization result in (1)~(4) and add it to the parameter setting, and grid search reg_alpha and reg_lambda two parameters;

6) Take the optimization result in (1)~(5) and add it to the parameter setting, and grid search learning_rate parameter.

## 4. Experiment

### 4.1 Data import

The data set collected in this paper contains biological activity values IC50 and pIC50 of compounds ERα, information on 729 molecular descriptors, interpretation of molecular descriptor meanings.

### 4.2 Data pre-processing

In this data set, IC50 is the biological activity value of the compound against ERα, which is an experimental measurement, where a smaller value represents greater biological activity and more effective in inhibiting ERα activity. The pIC50 is obtained by converting the IC50 value (i.e., the negative logarithm of the IC50 value), which usually has a positive correlation with biological activity, i.e., a higher pIC50 value indicates higher biological activity. In practical QSAR modeling, pIC50 is generally used to represent the bioactivity value. Variable selection is first performed for 729 molecular descriptors of 1974 compounds, and the top 20 molecular descriptors (i.e., variables) with the most significant effect on biological activity are selected by using a XGBoost-based data feature selection method to rank the variables according to their importance on biological activity.

### 4.3 XGBoost-based data feature selection

**Figure 1:**
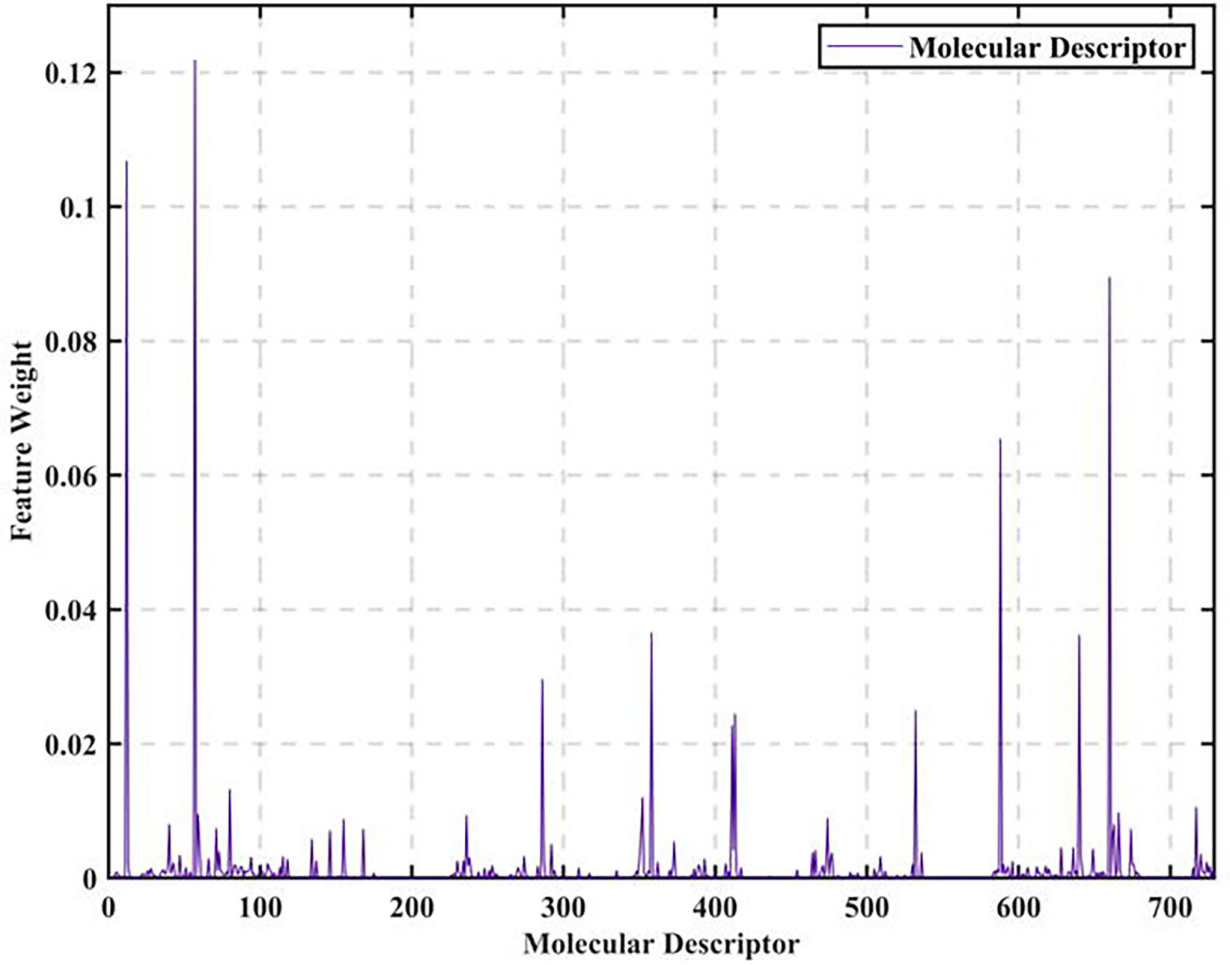
Molecular descriptor feature weights.

The data of 729 molecular descriptors of 1974 compounds are initially analyzed. We find that the data are of a certain scale and the influence factors obtained by adopting simple correlation analysis are not representative, so we adopt a more rigorous XGBoost-based data feature selection to calculate all 729 feature weights, as shown in **Figure 1**.

**Figure 2:**
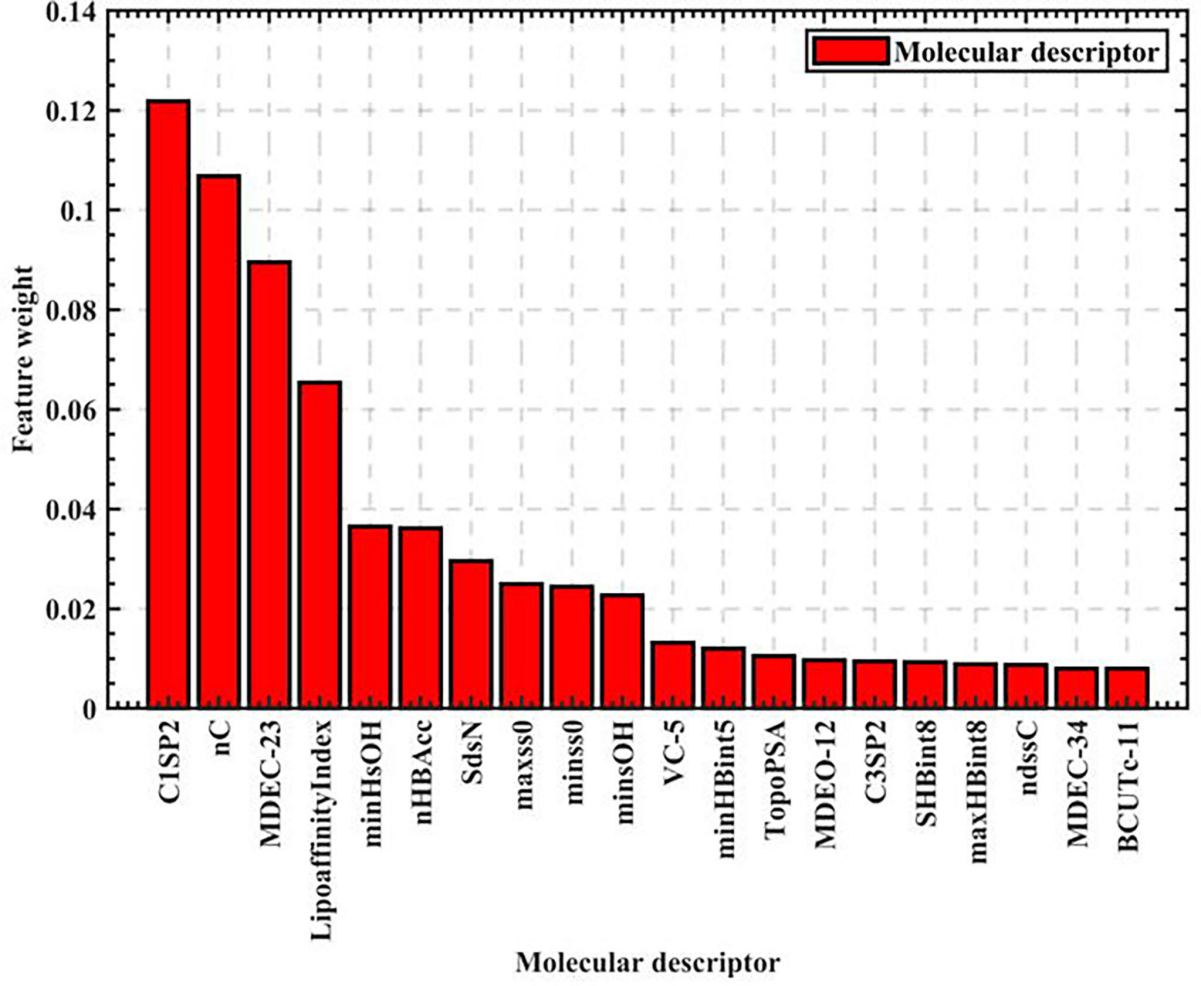
Top 20 ranking chart of feature weights.

As seen in **Figure 1**, the feature weights of the 729 molecular descriptors varies greatly overall, with the maximum weight exceeding 0.12 and the minimum weight close to 0. It is thus clear that the degree of influence of different molecular descriptors on biological activity varies greatly. Therefore, all the calculated molecular descriptor feature weights are output in descending order, and the top 20 molecular descriptors are intercepted as the most significant variables affecting biological activity, as shown in **Figure 2**.

The top 20 feature weights of 729 molecular descriptors of 1974 compounds are summarized according to **Figure 2**, which are ranked and summarized to finally obtain the top 20 molecular descriptors affecting biological activity, as shown in **Table 1**.

**Table 1:** Range of molecular descriptor values found by the optimization search model.

Molecular descriptors	Weighting value	Molecular descriptors	Weighting value
C1SP2	0.121828	VC-5	0.013187
nC	0.106756	minHBint5	0.011962
MDEC-23	0.089470	TopoPSA	0.010527
LipoaffinityIndex	0.065367	MDEO-12	0.009671
minHsOH	0.036505	C3SP2	0.009461
nHBAcc	0.036146	SHBint8	0.009285
SdsN	0.029560	maxHBint8	0.008846
maxss0	0.024947	ndssC	0.008720
minss0	0.024412	MDEC-34	0.007981
minsOH	0.022663	BCUTc-11	0.007961

### 4.4 Quantitative structure-activity relationship model

Based on the 20 molecular descriptors screened previously, we build the model according to the known data types by the four algorithms that have been selected. A total of 1974 sets of data exist in the data set, so we randomly select 50 compounds as the test set for IC50 values and corresponding pIC50 values prediction, and the remaining 1924 sets of data as the prediction set.

**Figure 3:**
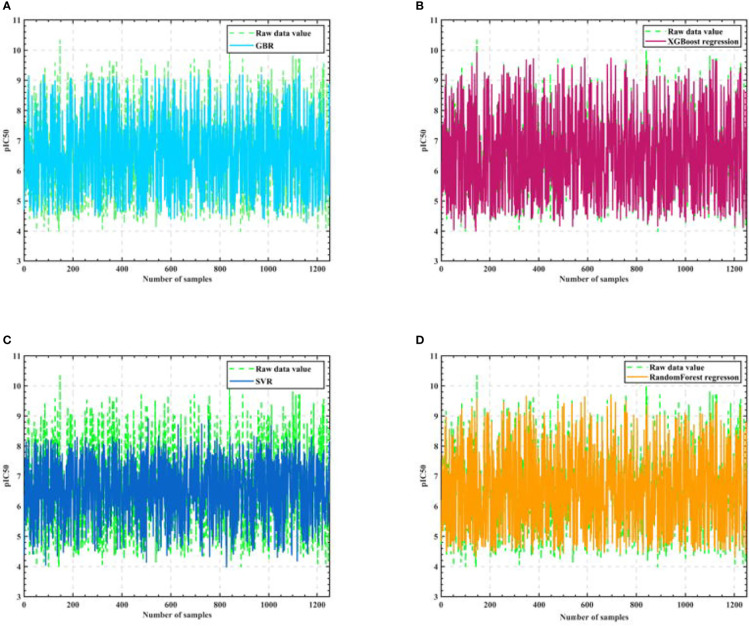
Algorithm model training process. **(A)** GBR **(B)** XGBoost regression **(C)** SVR **(D)** Random Forest regression.

Firstly, we eliminate the selected 50 compounds, and the remaining 1924 sets of compound data with the filtered 20 molecular descriptors as input and pIC50 values as output, use the cross validation method to segment the data with 0.35 as the sample ratio, so as to obtain the training set and test set, and then use the training set to train the Gradient-enhanced regression, XGBoost regression, Support vector machine and Random Forest regression models respectively, and the training process is shown in **Figure 3**.

As seen in **Figure 3**, the training results of Support vector machine are the worst, and the training results of Gradient-enhanced regression, XGBoost regression, and Random Forest regression models are better and do not differ much from each other. Therefore, we perform Semi-automatic parameter adjustment combining manual parameter adjustment and grid parameter adjustment on the three algorithm models of Gradient-enhanced regression, XGBoost regression and Random Forest regression to find the core algorithm.

**Figure 4:**
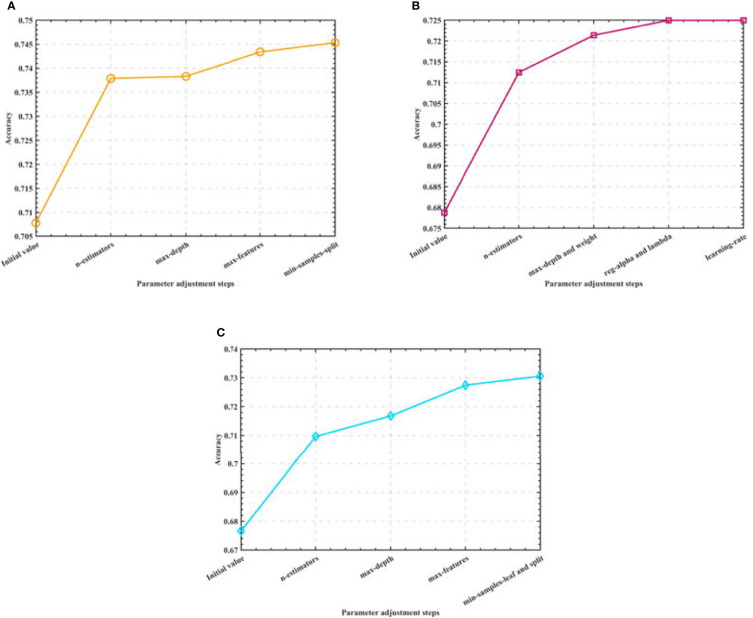
Semi-automatic parameter adjustment process. **(A)** Optimization process of Random Forest **(B)** Optimization process of XGBoost **(C)** Optimization process of GBR.

After that, we optimize the parameters of the three algorithms, and consider obtaining the model that is closest to the actual accuracy through the optimal parameters. Among them, in the Random Forest algorithm model, we optimize the number of trees, the maximum depth of trees, the maximum number of features, and the minimum number of samples allowed to split nodes. In the XGBoost algorithm model, we optimize the number of learners, the depth of the tree, the minimum weight of the subset, L1 regularization, L2 regularization and the learning rate. In the Gradient-enhanced algorithm model, we optimize the parameters of the maximum number of weak learners, the maximum depth of learners, the maximum number of features of learners, the minimum number of samples required by leaf nodes and the minimum number of samples divided into internal nodes. The process is shown in **Figure 4**.

By adjusting the parameters of the three algorithm models, we finally determine the optimal parameter combination of the Random Forest algorithm as: n_estimators=500, max_depth=90, max_features=0.1, min_samples_split=2; the optimal parameter combination of the XGBoost algorithm as: n_estimators=20, max_depth=7, min_child_weight=5, reg_alpha=1,reg_lambda=0.1, learning_rate=0.3; the optimal parameter combination of the Gradient-enhanced algorithm as: n_estimators=300, max_depth=4, max_features=0.3, min_samples_leaf=8,min_samples_split=9; and use the test set to test the algorithms before and after parameter adjustment. The test accuracy of the three algorithms is shown in **Table 2**.

**Table 2:** Comparison of accuracy before and after parameter adjustment.

Algorithm	Random Forest regression	XGBoost regression	GBR
Accuracy before parameter adjustment	0.707829	0.678772	0.676599
Accuracy after parameter adjustment	0.745329	0.724967	0.730603

In order to determine the core algorithm more accurately, we use the training data set and the test data set to train the above three parameter adjusted models, combined with a variety of regression model training error analysis methods to determine the algorithm with the best training effect. Among them, the regression model training error analysis methods we selected Mean Square Error (MSE), Mean Absolute Error (MAE), Root Mean Square Error (RMSE), Mean Absolute Percentage Error (MAPE), absolute coefficient (R-Square), and Explained Variance score (EV).

The model evaluation error analysis table, model testing error analysis table, and cross-validation results are shown in **Tables 3**, **4**.

**Table 3:** Model evaluation error analysis.

Evaluation Metrics	MSE	MAE	EV	R²
Random Forest	0.077646	0.203693	0.961473	0.961529
GBR	0.135665	0.268423	0.932684	0.932684
XGBoost	0.152522	0.290378	0.924355	0.924319

**Table 4:** Model test error analysis.

Evaluation Metrics	MAE	RMSE	MAPE	EV
Random Forest	0.544467	0.135665	0.089044	0.715558
GBR	0.557161	0.135665	0.090808	0.701300
XGBoost	0.567658	0.135665	0.091912	0.687493

After adjusting the error analysis of the model, it can be concluded that the Random Forest has the best accuracy and excellent anti over fitting, and the stability of the algorithm is high.

**Figure 5:**
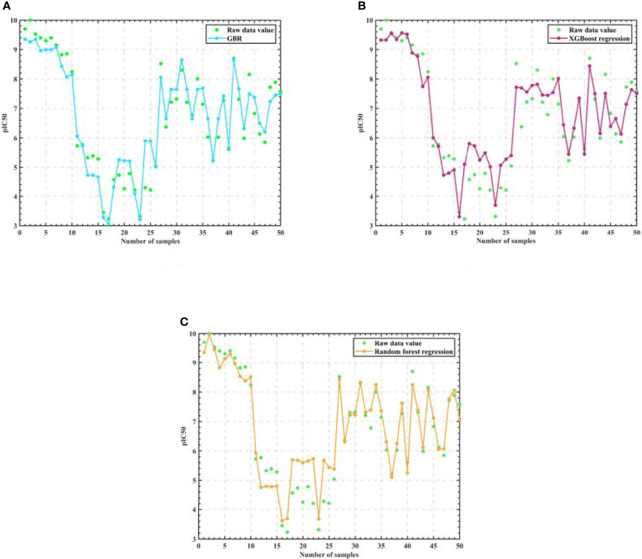
Algorithm model regression test results. **(A)** GBR **(B)** XGBoost regression **(C)** Random Forest regression.

Then, in order to verify that Random Forest is the best algorithm, we use three training and parameter adjusted algorithms: Random Forest, Gradient-enhanced and XGBoost to predict the IC50 value and the corresponding pIC50 value of the selected 50 groups of compound data, and the experimental results are shown in **Figure 5**.

**Figure 6:**
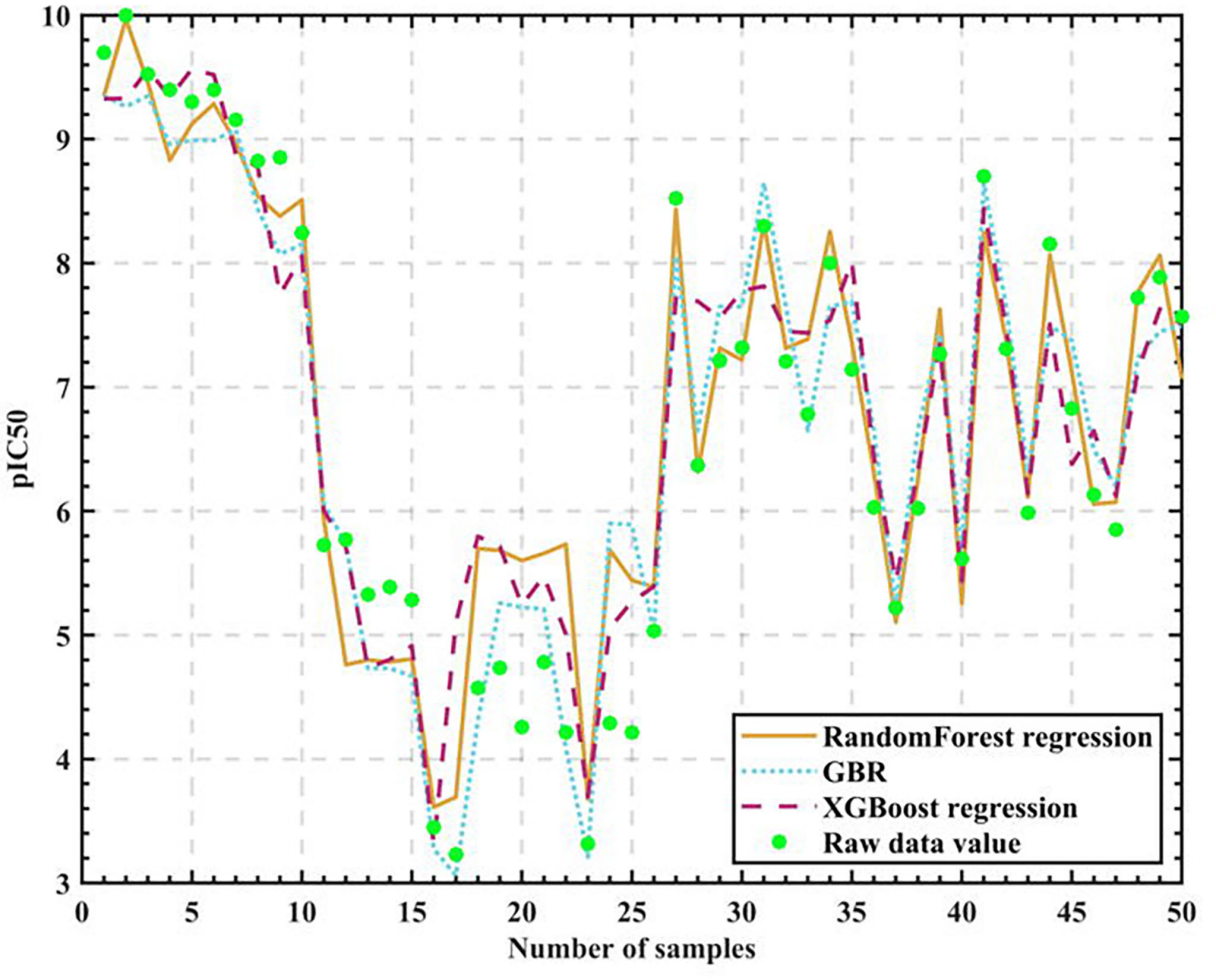
Algorithm model test results.

We experimentally compare the three regression algorithm models after tuning the parameters on 50 sets of test set data as in **Figure 6**.

We finally give the accuracy rates, as in **Table 5**.

**Table 5:** Final prediction accuracy.

Algorithm	Random Forest regression	GBR	XGBoost regression
Accuracy	0.766850	0.758679	0.756774

As can be seen from the table, Random Forest regression has the highest accuracy rate of 76.685%, so it can be considered reasonable for the Random Forest algorithm to be the core algorithm.

## 5 Conclusion

With the development of computer technology, in the early stage of anti-breast cancer drug research and development, using computer models to predict the biological activity of compounds is conducive to reducing the failure rate of drug research and development and saving a lot of research and development time and cost for research and development institutions. Therefore, in order to solve the problem of bioactivity prediction during the early development of anti-breast cancer candidate drugs, this paper has carried out relevant work and obtained the following conclusions.

(1) In this paper, we investigate compounds capable of antagonizing ERα activity by facilitating XGBoost-based data feature selection thereby screening the top 20 molecular descriptors with the most significant impact on biological activity.

(2) Then, from the perspective of molecular descriptors, with 20 molecular descriptors selected based on XGBoost feature as input and pIC50 value as output, multiple regression prediction models of Random Forest, Gradient-enhanced, XGBoost and SVM are constructed to predict ER biological activity. According to the degree of fitting between the predicted value and the real value, the Random Forest, Gradient-enhanced and XGBoost are preliminarily selected with good results. In order to select the best algorithm from the three algorithms, the Semi-automatic parameter adjustment method is used to adjust the parameters of the three algorithms. The Random Forest has the highest accuracy, the best accuracy and excellent anti over fitting, and the algorithm has high stability.

(3) Finally, by training the initial randomly selected data, it is verified that the Random Forest with Semi-automatic parameter adjustment has the best effect. It can be seen that using the Semi-automatic parameter adjusted Random Forest model to predict the bioactivity of compounds against breast cancer drugs can provide a good reference, and can play a certain role in promoting the optimization of drug properties in the process of cancer drug development.

The model proposed in this paper can provide better support for the early development of anti- breast cancer drugs, and the model can also be extended to other areas of prediction. However, the test accuracy of the model did not reach a particularly high level, so the subsequent optimization of drug properties for the problem will provide more solutions using artificial intelligence technology.

## Data availability statement

The original contributions presented in the study are included in the article/supplementary material. Further inquiries can be directed to the corresponding author.

## Author contributions

The first author did the experiment of this paper, and analyzed and compared the experimental results. The second author wrote this article, the third author edited the language of this article, and the fourth author and corresponding author put forward the idea of this article. All authors contributed to the article and approved the submitted version.

## Funding

This work was supported by: North China University of Science and Technology, Project Name : Research on Trusted Verification Technology of Cloud Outsourcing Computing, Project Number:0088/28415599.

## Conflict of interest

The authors declare that the research was conducted in the absence of any commercial or financial relationships that could be construed as a potential conflict of interest.

## Publisher’s note

All claims expressed in this article are solely those of the authors and do not necessarily represent those of their affiliated organizations, or those of the publisher, the editors and the reviewers. Any product that may be evaluated in this article, or claim that may be made by its manufacturer, is not guaranteed or endorsed by the publisher.
